# A preliminary antimicrobial investigation of lactobacillus bulgaricus probiotic supernatants as an intracanal medication against mature *E. faecalis* biofilm in an ex-vivo dentin blocks model

**DOI:** 10.1007/s00784-026-07125-x

**Published:** 2026-09-07

**Authors:** Sherif Heidar, Hayam Y. Hassan, Edgar Schäfer, Shehabeldin Saber

**Affiliations:** 1 Department of Endodontics, Faculty of Dentistry, Suez University, Suez, Egypt; 2 Department of Endodontics, Faculty of Dentistry, Suez Canal University, Ismailia, Egypt; 3 Department of Endodontics, Faculty of Dentistry, Badr University, Cairo, Egypt; 4 Central Interdisciplinary Ambulance, School of Dentistry, University of Münster, Waldeyerstr. 30, Münster, D-48149 Germany; 5 Department of Endodontics, Faculty of Dentistry, The British University in Egypt (BUE), El Sherouk City, Egypt; 6 Dental Science Research Group, Health Research Centre of Excellence (HRC), The British University in Egypt (BUE), El Sherouk City, Egypt

**Keywords:** Calcium hydroxide, *E. faecalis* biofilm, Intracanal medicament, *Lactobacillus bulgaricus*, Probiotics

## Abstract

**Aim:**

To provide a preliminary assessment of the antimicrobial potential of *Lactobacillus bulgaricus* probiotic against mature *Enterococcus faecalis* biofilm in dentin model under ex-vivo conditions using confocal laser scanning microscopy (CLSM).

**Methods:**

*Lactobacillus bulgaricus* and *Lactobacillus acidophilus* probiotic strains were grown, centrifuged, filtered by syringe filter size 0.45 microns to acquire cell free supernatants (CFS). Lyophilized CFS of each probiotic strain was individually mixed with Poloxamer gel using cold technique to prepare probiotic supernatants (PS) gel. Dentin blocks from cervical root portions were standardized at similar dimensions, infected with *E. faecalis* for 21 days to generate a mature biofilm. At the end of incubation, infected dentin blocks were randomly assigned to five groups (*n* = 10) based on the medication as follows: positive control (PC), calcium hydroxide (CH), *Lactobacillus acidophilus* PS gel (LA), *Lactobacillus bulgaricus* PS gel (LB), *Lactobacillus* combination PS gel (LC) combining LA and LB at a 1:1 ratio. All dentin blocks were medicated and incubated for 7 days under anaerobic conditions. Then, dentin blocks were examined with CLSM at 40X magnification; three-dimensional imaging in Z-stack mode was processed, and the dead *E. faecalis*% was quantitatively assessed using bioImage_L v21 software and total biovolume was calculated. Dead *E. faecalis*% were compared across groups using a one-way ANOVA test followed by Tukey’s post hoc test. Biovolume data were compared using the Kruskal-Wallis test followed by Dunn’s post hoc test with p-value adjustment using Holm’s method. The significance level was set at *p* < 0.05 for all tests. Statistical analysis was performed using R version 4.6.0 for Windows.

**Results:**

LB was linked to the highest percentage of dead *E. faecalis*% (*p* < 0.05), followed by LC, LA, CH, and the PC. Significant differences in dead *E. faecalis*% were observed between all groups (*p* < 0.05) except for CH and the PC groups. There was no significant difference in total biovolume among all groups.

**Conclusion:**

The Initial assessment of LB showed a promising antimicrobial action in dentin model that exceeded LA and CH. As biofilm biovolume was unaffected, LB appears to reduce bacterial viability rather than remove biofilm mass, warranting further validation in polymicrobial and intact canal models.

**Clinical significance:**

Probiotics may be of clinical relevance due to their antimicrobial action against *E. faecalis*.

## Introduction

Apical periodontitis is an inflammatory disease caused by a polymicrobial infection of the root canal system [1]. The primary goal of endodontic treatment is to eliminate this infection and prevent microbial re-invasion [2]. The disinfection phase is often challenged by the complex root canal anatomy. The presence of inaccessible regions within root canals offers shelter for the development, maturation, and establishment of bacterial biofilms [3], rendering shaping alone inadequate to clean canals [4]. Hence, the use of intracanal medications (ICMs) is recommended to reduce microbial load in infected root canal systems [5]. ICMs are also used during regenerative endodontic procedures as well as management of traumatic injuries and root resorptions [6]. CH is routinely used as an ICM owing to its alkalinity, clastic inhibition, and biomineralization [7]. However, CH controversial antimicrobial action against microbial biofilms especially *E. faecalis* [8], along its attenuated effect due to dentin buffering [9], and inability to penetrate infected dentinal tubules [10] represents drawbacks. Regenerative endodontic procedures address elimination of bacteria as a fourth pillar added to classic regenerative triad, despite proven antibacterial action of triple antibiotic pastes, several concerns are raised including antibiotic resistance, cytotoxicity and discoloration, maintaining CH as the most widely used ICM [11]. This advocates pursuing alternative ICMs.

Lilley and Stillwell in 1965 introduced the term “probiotics” to oppose the use of antibiotics, via beneficiary live bacteria [12]. The World Health Organization (WHO) defines probiotics as “live microorganisms which when administered in adequate amounts confer a health benefit on the host” [13, 14]. Probiotics antimicrobial action relies on competitive exclusion of pathogenic bacteria, releasing lactic acid, hydrogen peroxide, lipoteichoic acids and bacteriocin that collaborate to decrease pathogenic bacteria [15, 16].

Currently, probiotics are among innovative approaches due to their antimicrobial and anti-inflammatory effects, along with several advantages, including a high safety profile, affordability, and practical preparation [17, 18]. Beneficial outcomes associated with the use of probiotics have been reported in clinical studies of dental caries [19], gingivitis [20], and marginal periodontitis [21]. Although recent systematic reviews suggested the antimicrobial performance of probiotics in decreasing caries incidence and the instrumental adjunctive effects in periodontal therapy, evidence still inconclusive due to limitations in incorporated studies [22, 23].

There is an increased interest for probiotics in endodontics, due to antimicrobial action against endodontic pathogens [14, 21], release of biosurfactants that can exert an inhibitory effect on biofilm formation of root canals derived microorganisms under laboratory conditions [24], probiotics as an ICM can release transforming growth factors from the dentin matrix opening new horizon for usage in regenerative endodontic procedures [25], systemic administration of probiotics reduced apical periodontitis severity in rats [26, 27].

*Lactobacillus* probiotics are among most commonly used probiotics [21], owing to its antagonizing effects against a broad range of human pathogens and being included among Generally Recognized As Safe (GRAS) substances [13]. To date, few probiotics strains have been investigated in gel form to serve as an ICM. Laboratory tests were done to evaluate the antimicrobial effects of *Lactobacillus acidophilus*, *Lactobacillus rhamnosus*, *Lactobacillus plantarum*,* Lactobacillus Casei*,* Bifidobacterium bifidum* against *E. faecalis* and Candida albicans showed promising results [13, 15]. A CLSM analysis on glass model demonstrated that *Lactobacillus* lipoteichoic acids inhibit *E. faecalis* biofilm formation [16]. However, the aforementioned studies lack the assessment on dentin, that underweights the concluded promising pattern of probiotics as disinfection strategy.

*E*. *faecalis* biofilm continues to be meaningful for initial screening of a new disinfection strategy, to provide insights into antimicrobial mechanism [28]. Human dentin is the favorable substrate for studying ICMs [29]. Endodontic literature encompasses 2 studies of probiotics on dentin and promising results were stated on both studies. The scanning electron microscope (SEM) study demonstrated that *Lactobacillus* probiotics inhibited *E. faecalis* biofilm formation on dentin [16] although, SEM is qualitative two-dimensional assessment. A culture analysis study showed that PS performed microbial reduction in a tooth model, although the quantification methodology is highly subjective [14]. Yet, antimicrobial action of PS in dentin still needs further investigations, as only few available data are currently available in the literature and heterogenicity of existing studies. The validity of dosage and application still lack verified consensus.

The current study is the first study in endodontics to implement the usage of LB as an antimicrobial using a dentin model. *Lactobacillus bulgaricus* has a broad-spectrum antimicrobial, anti-inflammatory, colitis-associated cancer inhibitor and wound-healing potentials [30]. The current study is the first study to employ CLSM, as sensitive quantitative approach to investigate the antimicrobial effectiveness of PS as potential ICMs against an ex-vivo dentin model. The null hypothesis proposed that there would be no significant difference between *Lactobacillus bulgaricus*, alone or in combination with other probiotic strain against the gold standard ICM, calcium hydroxide.

## Materials and methods

This study was approved by the Research Ethics Committee (Approval No. 25–031 on 1/8/2025) at the British University in Egypt. All procedures were carried out in accordance with relevant guidelines and regulations. In the present study the examiner who performed the confocal laser scanning microscopy (CLSM) and did the imaging and analysis of the samples, as well as the statistician were blinded to the experimental groups.

### Probiotic preparation

Two *Lactobacilli* Probiotics strains, *Lactobacillus acidophilus* and *Lactobacillus bulgaricus*, were purchased from Apex Biotechnol, Indore-Madhya Pradesh, India. Each strain was aseptically grown in a flask containing 100 mL De Man, Rogosa, and Sharpe (MRS) broth (HiMedia Laboratories Pvt. Ltd, Mumbai, India) under anaerobic conditions at 37 °C for 72 h. Then submitted for centrifuge (Microcentrifuges, ThermoFisher Scientific, Cleveland, USA) for 10 min at 10,000 rpm, filtered by syringe filter size 0.45 micron (*MilliPore*, *Darmstadt*,* Germany*) to acquire the cell-free supernatant (CFS) and finally lyophilized using a vacuum freeze dryer (Model FDF 0350, Korea) as previously described [13, 14].

### Poloxamer gel preparation

Poloxamer was selected for gel preparation based on results of previous studies as an ideal vehicle for PS gel [15] and favorable performance reported [13, 14]. Cold technique was used for Poloxamer gel preparation needed for ICM, Poloxamer 407 powder (Sigma Aldrich Chemical, Gillingham, UK) was gradually added to cold water (4–8 °C) under magnetic stirrer (Scilogex, Rocky Hill, CT, USA) up to a final concentration of 30% (w/w) and kept in refrigerator for 24 h as previously described [13, 14].

### Probiotics supernatants (PS) gel

Lyophilized CFS of each probiotic strain was individually mixed with Poloxamer gel to obtain *Lactobacillus acidophilus* PS gel (LA), *Lactobacillus bulgaricus* PS gel (LB). *Lactobacillus* cocktail PS gel (LC) incorporated both lyophilized CFS at ratio (1:1). Concentration of all PS gel was set at 300 mg lyophilized CFS/ 1 mL Poloxamer gel. This concentration was selected according to results of a previous study demonstrating that PS concentrations of 300 mg/mL showed an optimal antimicrobial performance [14].

### Sample size calculation

A power analysis was conducted to ensure sufficient power to detect a significant difference in antibacterial efficacy among the tested groups. Using an alpha (α) level of (0.05), a beta (β) level of (0.2) corresponding to (80%) statistical power and effect size (Cohen’s f) of (1.16) derived from the results of a previous study by Ferrao et al. [31]; the minimum total required sample size (n) was found to be (15) samples (3 per group). The sample size was increased to account for possible variability and to increase the robustness of statical testing to 65 samples (10 per group and 5 for validation of biofilm maturation). The sample size was calculated using R statistical analysis software version 4.6.0 for Windows [32].

### Human teeth collection

Human single rooted mature teeth, recently extracted due to periodontal diseases from young age patients (20–30 years), were collected. All attached plaque, calculus and soft tissues were removed using surgical scalpel. All teeth were imaged using periapical radiograph (Xgenus DC, de Gotzen, Fagnano Olona, Italy) twice from buccal and mesial aspect to include only teeth with mature apex, Vertucci type I root canal configuration, and patent pulp space. All teeth with pulp canal calcifications, resorptive defects, morphological anomalies, previous retrograde fillings and open apices were excluded. Teeth were inspected under dental operating microscope (Leica M320, Leica, Mannheim, Germany) to exclude teeth with caries, cracks, and previous restorative/pulpal interventions. Finally, a total number of 32 teeth were sterilized then stored in a 0.5% thymol solution at 4 °C and used within one month of storage.

### Dentin blocks preparation

Flat dentin surfaces were required to overcome CLSM limited depth of view [33]. Consequently, the dentin blocks preparation followed the methodology described by Ruiz-Linares et al. [34], the crowns, middle and apical root portions of teeth were sectioned and discarded. Afterwards, the coronal root portions were divided longitudinally into two halves. Then, a 220 to 800 grit silicon carbide papers (3 M, St. Paul, MN, USA) were used to polish inner surfaces of root canals to obtain almost flat surfaces, then the cementum and outer dentin were removed to obtain 64 dentin blocks. Each dentin block was adjusted by a caliper to exact dimensions 4 × 4 × 0.7 mm (width x length x height).

Dentin blocks were submerged in 17% EDTA (Endo-Solution, Cerkamed, Stalowa Wola, Poland) ultrasonic bath for four minutes to remove smear layer, then irrigated with 5 mL sodium hypochlorite (Chloraxid 2%, Cerkamed, Stalowa Wola, Poland). Afterwards, dentin blocks were rinsed with distilled water for 10 min and autoclaved for 20 min at 121 °C and pressure 15 psi [31]. To guarantee sterility, dentin blocks were rehydrated on Brain Heart Infusion (BHI) broth medium (Oxoid, Basingstoke, UK) and incubated at 37 °C for 2 days, sterility was verified by absence of turbidity.

### Classification of the samples

The discs were randomly divided into six groups (*n* = 10) according to the ICM, as follows: negative control (non-infected, non-medicated), positive control (infected, non-medicated), and four experimental groups medicated with either CH (UltraCal XS, Ultradent, South Jordan, UT, USA), LA, LB and LC.

### *E. faecalis* biofilm generation

Dentin blocks from the positive control and experimental groups were infected following the methodology previously described by Bukhari et al. [35], *E. faecalis* (ATCC 29212) colonies were grown in blood agar (Oxoid Basingstoke, UK), then *E. faecalis* pure colonies were suspended in BHI medium and incubated overnight, then bacterial suspension was adjusted spectrophotometrically at 600 nm optical density. In 24 well plate (Olympus 24 well plate, 3.5 mL; Genesee Scientific, San Diego, CA), each dentin block was transferred to a well containing 2 ml sterile BHI medium, and finally 500 µL of the bacterial suspension was added to each well. All dentin blocks in the wells were then incubated for 21 days at 37 °C under anaerobic conditions to generate *E. faecalis* biofilm in dentin blocks. BHI Medium was replenished every 2 days.

### Biofilm verification on dentin at the end of infection period using CLSM

At the end of 21 days incubation in BHI media, 5 dentin blocks were submitted for CLSM to verify the biofilm maturation prior to medication as previously mentioned [31, 36]. Each dentin block was rinsed with 5 mL of distilled water to remove remaining broth media, stained in a dark room using 30 µL of the Live/Dead BacLight bacterial viability kit (Invitrogen Molecular Probes, Eugene, OR, USA) containing 1:1 mixture of Syto 9 and propidium iodide (PI) for 20 min, Syto 9, a green fluorescent dye that stains live bacteria green, and PI is a red fluorescent dye that stains dead bacteria red [37]. Following staining, dentin blocks were then rinsed by saline, mounted, and directly investigated by CLSM (Leica DMi8, Leica, Mannheim, Germany). The scanning was performed from the top of the biofilm towards dentin surface [37].

### Dentin blocks medication

Each dentin block was flushed by 5 mL distilled water to remove planktonic *E. faecalis* and residual media, then fixed in Petri dishes. Dentin blocks of the experimental groups were treated with assigned medications following the methodology previously described by Freitas et al. [38]. Each dentin block was coated with 0.1 mL of tested ICM for treatment groups/saline for control groups, then covered by sterile cotton soaked with distilled water to produce 100% humidity and incubated at 37 °C for 7 days under anaerobic conditions. After accomplishing the designated ICM employment period, each dentin block was irrigated with 1mL of appropriate neutralizing solution: citric acid (Cerkamed, Stalowa Wola, Poland) adjusted at a concentration of 0.5% for the CH group and saline 0.9% for all other groups.

### Confocal laser scanning microscopy imaging for treatment and control groups

Each dentin block was rinsed with 5 mL of distilled water, stained with Syto 9 and PI fluorescent dyes and directly investigated by CLSM. Scanning was performed using a 40X oil lens at a 1024 × 1024-pixel format. Syto 9 and PI fluorescent dyes were excited with 488 and 532 nm wavelengths, with emission detected between 490 and 575 nm for green fluorescence and between 600 and 720 nm for red fluorescence [39]. In each dentin block, five random regions were selected for three-dimensional (3D) imaging in Z-stack mode, covering an area of 220 × 220 μm with a 1 μm step size from the superficial part of the *E. faecalis* biofilm to the deeper dentin. Sequential images were then processed with Leica Application Suite X software (LAS X, Leica), and the dead *E. faecalis*% and total biovolume (µm^3^) were quantified using bioImage_L v21 software [40].

### Statistical analysis

Continuous data were presented as mean ± standard deviation (SD), median, and interquartile range (IQR). The data were assessed for normality by viewing the distribution and using the Shapiro-Wilk test. Variance homogeneity was assessed using Levene’s test. The *E. faecalis*% did not deviate substantially from either assumption; however, the total biovolume data were not normally distributed. Dead bacterial cells were compared across groups using a one-way ANOVA test followed by Tukey’s post hoc test. The model was assessed for residual normality using methods described earlier, with no deviation. Total biovolume data were compared using the Kruskal-Wallis test followed by Dunn’s post hoc test with p-value adjustment using Holm’s method. The significance level was set at *p* < 0.05 for all tests. Effect sizes were interpreted according to Cohen 1992 [37]. Statistical analysis was performed using R version 4.6.0 for Windows [29].

## Results

**Fig. 1:**
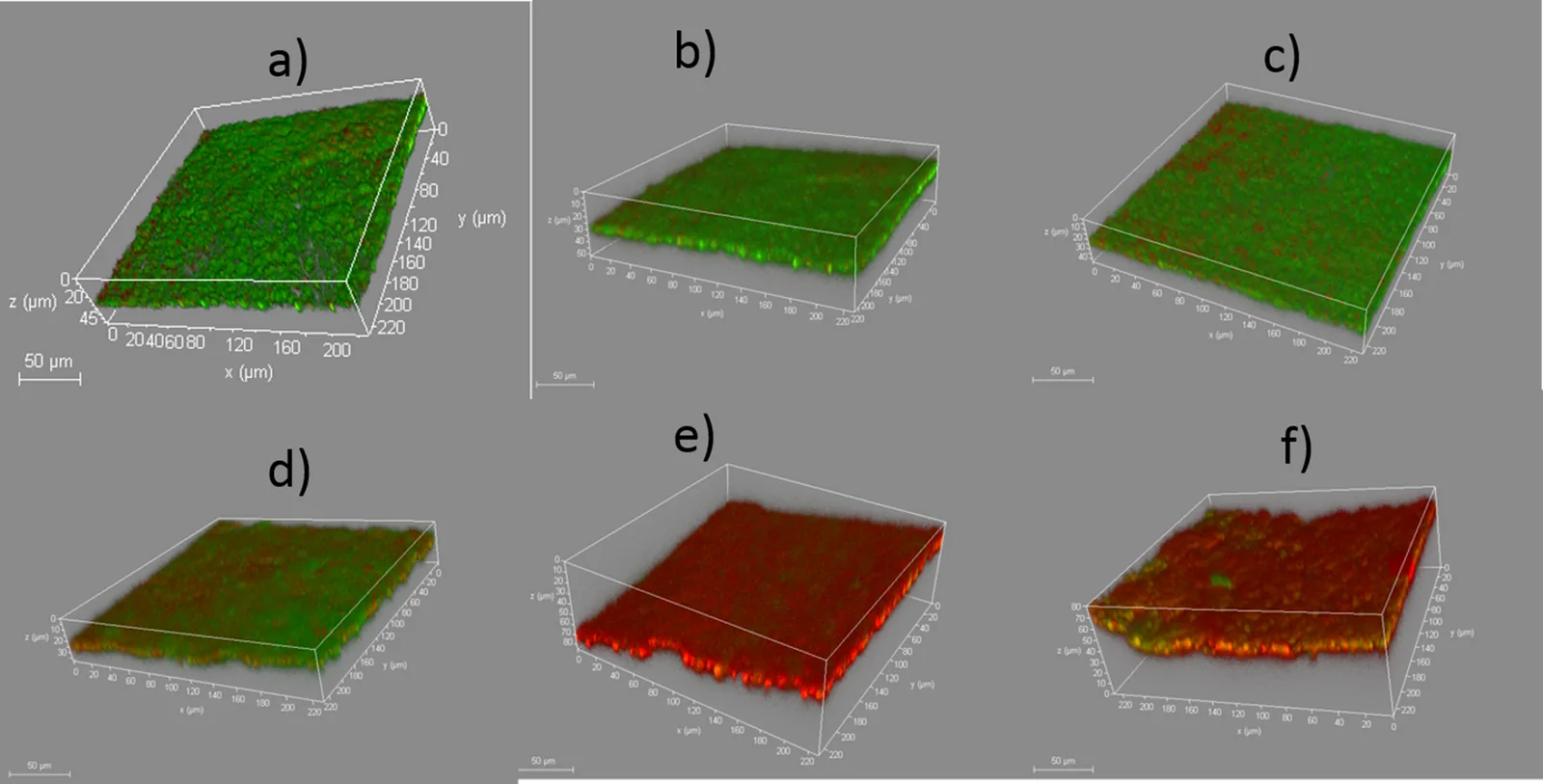
Representative 3D imaging of *E. faecalis* biofilm in dentin blocks obtained by integrating sequential 1 μm step size of Z-stacks using Leica software. (**a**) demonstrating biofilm verification at 21 days of incubation, (**b**) demonstrating dentin block from PC group, (**c**) representative images of CH group, (**d**) representative images of LA group, representative images of LB group and f) representative images of LC group. Higher red intensity was detected in LB, followed by LA, LC, respectively

The mature biofilm formation was verified using 3D images captured by CLSM, evident by dense bacterial aggregations (Fig. 1a).

The CLSM image analysis of the negative control group showed no evidence of *E. faecalis* biofilm in dentin blocks and accordingly *E. faecalis*% recorded was 0.

According to CLSM images, PC group demonstrated an increased green fluorescence denoting viable *E. faecalis* in the biofilm (Fig. 1b). The PC group showed the lowest dead *E. faecalis*% among all groups (Table 1).

**Table 1:** Dead *E. faecalis*% statistics summary and intergroup comparisons

	Positive control (infected/non-medicated)	Calcium hydroxide	Lactobacillus acidophilus probiotic supernatants	Lactobacillus bulgaricus probiotic supernatants	Lactobacillus combination probiotic supernatants	p-value	Effect size (95% CI)**
Mean ± SD	5.38 ± 2.93^D^	5.53 ± 2.39^D^	45.46 ± 8.17^C^	84.51 ± 7.09^A^	68.40 ± 8.05^B^	< 0.001*	**0.97 (0.95 to 0.98)**
Median (IQR)	4.38 (3.59)^D^	5.46 (2.50)^D^	48.68 (13.07)^C^	85.54 (6.73)^A^	68.24 (9.54)^B^		

** Partial eta squared for dead bacterial cells, *CI* confidence interval, values with different superscripts within the same horizontal row are significantly different, * significant (*p* < 0.05)

In CH group, the CLSM images revealed the abundance of green fluorescence, signifying the abundance of live *E. faecalis* (Fig. 1c). Statistical analysis showed no statistically significant difference between group CH and PC group (*p* > 0.05) (Table 1).

The CLSM images of the LA group demonstrated a higher dead *E. faecalis*%, as evidenced by increased red intensity compared to both CH and PC groups (Fig. 1d). Contrastingly to CH, pairwise comparison demonstrated that LA group exhibited a significantly higher dead *E. faecalis*% compared to both PC and CH groups (*p* < 0.05) (Table 1).

Dentin blocks medicated with LB showed the greatest antimicrobial activity, as evidenced by the highest red fluorescence intensity (Fig. 1e). According to statistical analysis, the LB group demonstrated significantly the highest dead *E. faecalis*% among all groups (*p* < 0.05) (Table 1).

In LC group, CLSM images revealed greater red fluorescence intensity than LA, CH and PC (Fig. 1f). however, LC group demonstrated lower red fluorescence intensity compared to LB group (Fig. 1). Statistical analysis demonstrated that dead *E. faecalis*% was significantly higher than the LA group (*p* < 0.05) but lower than LB group (*p* > 0.05) (Table 1).

In conclusion, pairwise comparisons revealed that LB had significantly the highest dead *E. faecalis*% values among all groups (*p* < 0.05). Additionally, the LC group also showed significantly higher dead *E. faecalis*% values than the remaining groups (*p* < 0.05), further intensifying the effective action of LB when used combined with LA. Finally, the LA group had significantly higher dead *E. faecalis*% values than the CH and PC groups (*p* < 0.05), while the difference between the latter two groups was not statistically significant (*p* > 0.05) (Table 1).

**Fig. 2:**
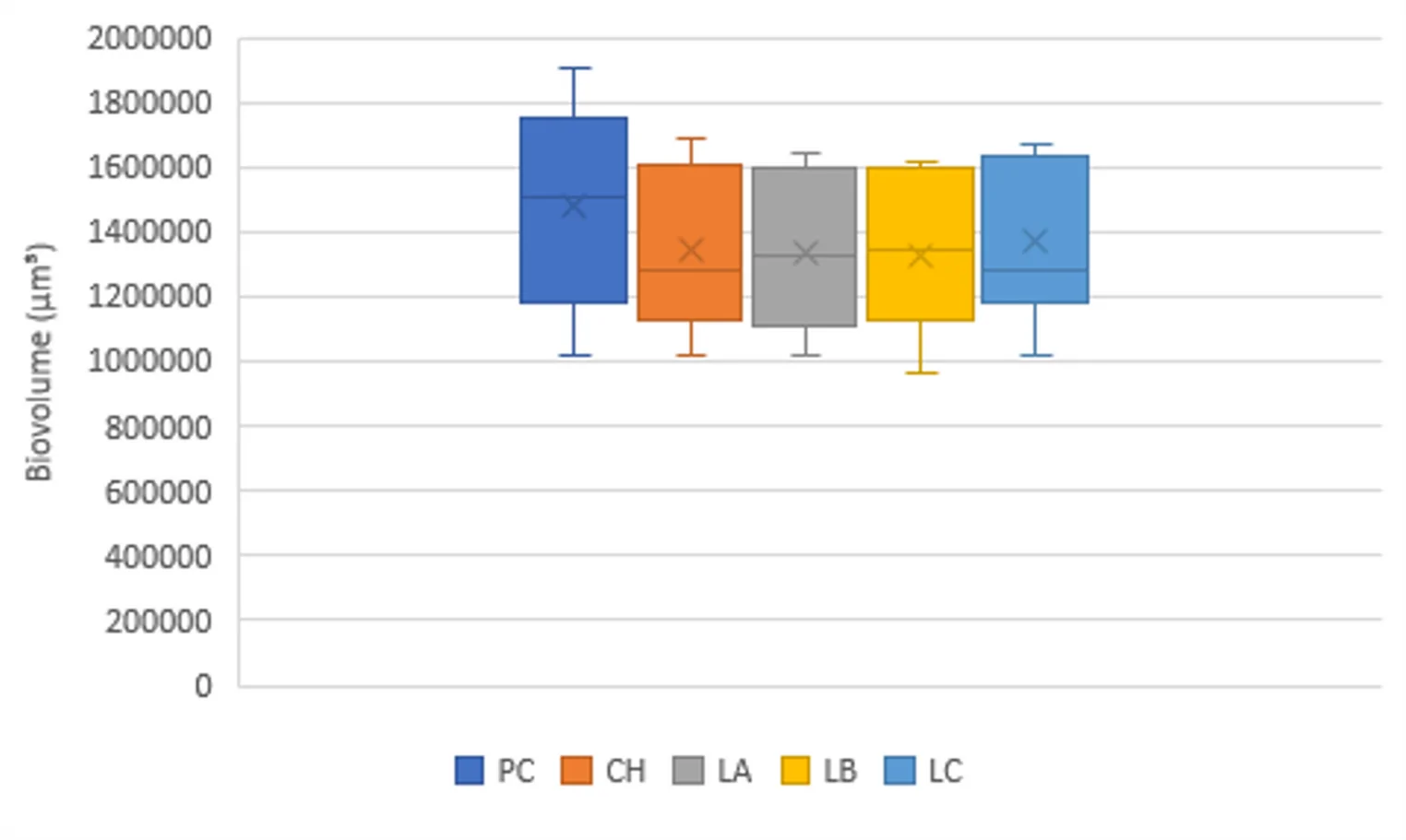
Box plot showing biovolume values in different groups

The total biovolume was calculated for all groups. Statistical analysis showed no significant difference between LA, LB, LC, CH and PC (*p* > 0.05) (Fig. 2).

## Discussion

The current study represents a preliminary assessment of LB as a novel probiotic strain, used alone in combination with LA, as a potential alternative to currently available ICMs. To provide a reliable assessment of *Lactobacillus bulgaricus* potentials to enhance available ICMs, the study included *Lactobacillus acidophilus* and calcium hydroxide as being extensively investigated, to set relevant comparison of ICM under investigation with ICMs with proven antimicrobial action. The combination of probiotic strains (LC) in the current study was previously mentioned by Shaaban et al. but with different strains [14].

Since the action of antimicrobials is significantly reduced by dentin buffering [33, 36], human dentin was chosen as the substrate of choice in the present study. Human dentin blocks were used to investigate the ex vivo antimicrobial action as suggested by Haapasalo & Ørstavik [41]. The blocks were obtained from the cervical thirds of young teeth to avoid sclerotic dentin that hinder bacterial invasion of dentinal tubules and diffusion of antimicrobials [33]. This model was chosen to align with CLSM microscopy, which has an inherent optical imaging-depth ceiling: reliable resolution of bacterial viability within dentinal tubules requires a flat, polished, optically accessible surface positioned within the objective’s working distance. An intact, curved, tapering root canal cannot be imaged in this way without physical sectioning, and unsectioned or unpolished dentin surfaces produce substantially degraded fluorescence signal due to light scattering by the mineralized substrate [42]. Beyond this optical constraint, standardized dentin blocks allow the control of variables that are otherwise major confounders in whole-root models, including canal curvature, taper, cross-sectional geometry, and the coronal-to-apical gradient in dentinal tubule density, all of which vary substantially between and within extracted teeth and would have made it difficult to attribute antibacterial differences specifically to the ICM under study rather than to uncontrolled anatomical variation. The block model further enabled standardized bacterial inoculation, uniform medicament contact area and diffusion path length, and a reproducible, position-matched imaging protocol across all specimens and treatment groups. Yet, future validation in an intact single- or multi-rooted canal model, and ultimately in a clinical setting, represents an important next step to confirm whether the antibacterial trends observed here can be translated to the more complex three-dimensional canal environment [43].

Although multispecies biofilm is more difficult to eradicate and represent a more clinically relevant rigorous biofilm, the mono-species *E*. *faecalis* biofilm continues to be meaningful in experimental setup for screening a new disinfection strategy to provide insights into antimicrobial mechanisms avoiding complexity, technical demands and time-constraints [28]. Mono-species *E. faecalis* biofilm was selected as a test microorganism in the current study, as tested ICMs include *Lactobacillus bulgaricus* as a new antimicrobial. *E. faecalis* (ATCC29212) is the most frequently investigated microorganism in disinfection studies [33] and accordingly considered the reference type for preliminary studies as it can provide reliable comparisons with available results in the literature [44].

Biofilm maturation was confirmed at the end of 21 days incubation in BHI media, using CLSM as previously mentioned [31, 36]. This method permits 3D visualization of the biofilm and provided a consistent reference for comparison, as all groups were examined under identical CLSM conditions.

Poloxamer 407, prepared using the cold technique, was selected as delivery vehicle for probiotic supernatants, owing to its inverse thermosensitivity behavior, it is soluble in aqueous solutions at temperatures below 4 °C, however, but forms a gel upon warming [15]. This choice was advocated by its performance on previous employing probiotic supernatants [13, 14]. Based on the results of a prior study comparing several PS concentrations, a PS concentration of 300 mg/mL was associated with the most favorable antimicrobial performance [14].

Results of the current study demonstrated the superior antimicrobial effect of PS compared to CH. Although it is the first time to evaluate *Lactobacillus bulgaricus* in an endodontic context, LB exhibited the most pronounced antimicrobial action among all tested groups, highlighting its potential for future endodontic research. This finding aligns with the observations of Bohora and Kokate [15] and Shaaban et al. [13]. *Lactobacillus* PS, specifically LA, have proven ability to perform antibiofilm effects against *E. faecalis* biofilms in dentin blocks via lipoteichoic acid production that inhibits extracellular polymeric substances (EPS) production that constitutes the biofilm, controlling biofilm-related genes and breaking down EPS [16]. Contradictory results were reported by Shaaban et al. who concluded that both PS and CH are effective antimicrobials on dentin, however CH was more effective than PS [14]. This discrepancy may be attributed to culture-based methodology inaccuracies and sampling method included during their study, probably paper point/dentin sampling did not include *E. faecalis* inside dentinal tubules or in areas inaccessible for files, which may cause misleading results. The CLSM technique provides more reliable results, as it represents direct approach, overcoming the sampling limitations.

The results also demonstrated that the LC medicated group showed an intermediate action between LA and LB. This finding can be attributed to reduced LB concentration, which presented the highest antimicrobial action. This result suggests a lack of synergism between the two probiotic strains when combined.

According to results of the current study, there was no significant differences between CH and PC group in dead *E. faecalis*%. This result is in alignment with Omer et al. [45], Siqueira and Uzeda [46], who reported that CH lacks antimicrobial effects against *E. faecalis*, although CH upon mixing with liquid dissociates into calcium and hydroxyl ions that elevates PH to (12.5–12.8), alkalinity can cause denaturalization of bacterial proteins, DNA and cytoplasmic membrane, but *E. faecalis* proton pump action probably acidifies the environment and forms biofilms in dentin beside dentin buffering capacity that may reduce the pH below lethal levels to *E. faecalis*. Antimicrobial action of CH against microbial biofilms is deemed controversial and less effective against *E. faecalis* [8, 47]. Moreover, the limited diffusion of CH in dentinal tubules probably reduce its action on *E. faecalis*, that invades dentinal tubules [48]. However, results of the current study are in disagreement with those of Lima et al., who reported that CH in all combinations was associated with bacterial reduction in dentin compared to control group [49].

The results further demonstrated no statistically significant difference in total biovolume among all groups. Although Jung et al. reported that probiotics can perform antibiofilm action by reducing extracellular polymeric substances that constitutes the biofilm [16], the ICM in the current study were applied after biofilm maturation in dentin blocks; accordingly, that inhibitory effect might not have been operative under the present experimental conditions.

The preliminary nature of the current study is associated with some limitations, which should be taken into consideration. Although dentin is a favorable substrate, age-related deposition of peritubular dentin may reduce the permeability and the number of infected dentinal tubules, variability in dentin mineralization also cannot be fully controlled and may confound the results [33]. Dentin blocks lack the root canal anatomical challenges that hinders the proper diffusion of antimicrobials, although provide a good starting point for preliminary investigation of LB [43]. Future efforts are recommended to transform the investigated model into a root canal configuration. Hence, whether and, if so, to what extent the antimicrobial effect of the tested ICMs might be negatively affected by organic material inside the root canal remains open to question, as usually organic matter decreases the efficiency of most disinfectants.

The CLSM methodology used in the present study provided in situ visualization and quantification of a biofilm. However, beyond the requirement of flat dentin surfaces, the high magnification employed limits imaging to a few randomly selected fields, as scanning the entire block is not feasible [33]. Molecular analysis using next generation sequencing techniques are recommended to complement CLSM assessment in future studies. Furthermore, a mono-species *E. faecalis* biofilm was used, as this approach is widely accepted for initial assessment of new ICM [28]. Nevertheless, this can also be considered as a limitation of this study and should be addressed in future work by using ex-vivo multi-species biofilms or by conducting clinical trials [50].

Heterogenicity of available studies in terms of probiotics usage, strains variation, dosage, methodological quality and preparation methods underscores that probiotics remain under investigation for dental application rather than being universally accepted for clinical use [51]. In addition, given the novelty of LB in this context, the present findings are not yet supported by sufficient evidence from longitudinal studies in the literature [22, 23]. Worth mentioning, the probiotics tested in the current study lack the radiopacity essential for clinical applications, and the addition of radio-opacifiers may negatively impact their antimicrobial and biological properties. Finally, the present results do not permit any conclusions regarding potential beneficial effects of the tested probiotics on endotoxins, an aspect that warrants further investigations.

## Conclusions

Within the limits of this preliminary ex vivo study, *Lactobacillus bulgaricus* demonstrated promising antimicrobial action against *E. faecalis* biofilm in a dentin substrate model, outperforming the other tested intracanal medicaments. The probiotic supernatants evaluated in the current study proved to be potent antimicrobial agents, capable of overcoming the antibiofilm limitations that continue to compromise the clinical performance of calcium hydroxide. Nonetheless, sufficient evidence has not yet been established in the literature to support the clinical use of *Lactobacillus bulgaricus* in daily endodontic practice. Future studies should focus on *Lactobacillus bulgaricus*, following a more comprehensive experimental design that incorporates multi-species biofilm models, intact root canal systems, and longitudinal clinical validation.

## Data Availability

Available upon reasonable request from the corresponding author.
